# A Novel Region-Growing Based Semi-Automatic Segmentation Protocol for Three-Dimensional Condylar Reconstruction Using Cone Beam Computed Tomography (CBCT)

**DOI:** 10.1371/journal.pone.0111126

**Published:** 2014-11-17

**Authors:** Tong Xi, Ruud Schreurs, Wout J. Heerink, Stefaan J. Bergé, Thomas J. J. Maal

**Affiliations:** 1 Department of Oral and Maxillofacial Surgery, Radboud University Nijmegen Medical Centre, Nijmegen, the Netherlands; 2 Department of Radiology, University Medical Centre Groningen, Groningen, the Netherlands; University of Toronto, Canada

## Abstract

**Objective:**

To present and validate a semi-automatic segmentation protocol to enable an accurate 3D reconstruction of the mandibular condyles using cone beam computed tomography (CBCT).

**Materials and Methods:**

Approval from the regional medical ethics review board was obtained for this study. Bilateral mandibular condyles in ten CBCT datasets of patients were segmented using the currently proposed semi-automatic segmentation protocol. This segmentation protocol combined 3D region-growing and local thresholding algorithms. The segmentation of a total of twenty condyles was performed by two observers. The Dice-coefficient and distance map calculations were used to evaluate the accuracy and reproducibility of the segmented and 3D rendered condyles.

**Results:**

The mean inter-observer Dice-coefficient was 0.98 (range [0.95–0.99]). An average 90^th^ percentile distance of 0.32 mm was found, indicating an excellent inter-observer similarity of the segmented and 3D rendered condyles. No systematic errors were observed in the currently proposed segmentation protocol.

**Conclusion:**

The novel semi-automated segmentation protocol is an accurate and reproducible tool to segment and render condyles in 3D. The implementation of this protocol in the clinical practice allows the CBCT to be used as an imaging modality for the quantitative analysis of condylar morphology.

## Introduction

Morphologic changes of jaw joints, or condyles, in form of condylar remodeling and condylar resorption are observed regularly following orthognathic surgery [1]–[4]. In contrast to the self-limiting, physiologic form of condylar remodeling, progressive pathologic condylar resorption (PCR) is an irreversible process that is characterized by severe dimensional changes of the condylar head and reduction of the posterior facial height [1], [2]. PCR is associated with late postoperative relapse, and may compromise the aesthetic and functional result of orthognathic surgery [2], [4]. In order to evaluate the disease progression and subsequent skeletal changes, radiographic assessment of condylar morphology is required.

In the past decade, cone-beam computed tomography (CBCT) has become a well-established imaging technique within oral and maxillofacial surgery. In contrast to the fan beamed x-ray used in conventional CT, a cone shaped x-ray beam is used in CBCT, allowing the acquisition of a real-size dataset based on a single low-radiation-dose scan, with the potential of scanning the patient in an upright position [5]. The CBCT is an alternative to conventional two-dimensional (2D) (e.g. orthopantomogram and lateral cephalogram) and three-dimensional (3D) (e.g. spiral CT) imaging modalities, providing 3D image data of hard and soft tissue structures. 3D virtual head models of the soft and bony tissue of the maxillofacial region, including the condyles, can be rendered from CBCT data sets for accurate diagnosis, predictable virtual surgical planning, postoperative follow-up and patient education [6], [7].

**Figure 1:**
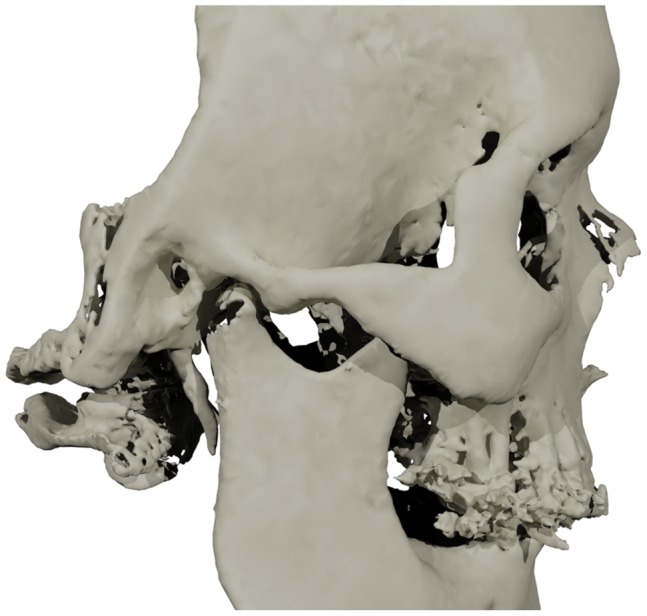
A 3D rendered virtual head model of a patient from Maxilim, reconstructed from the original CBCT data. The inaccurate reconstruction of the condyle is clearly visible. No measurement of the condylar shape or volume can be made due to the discontinuity of the condylar surface.

Despite the obvious advantages of 3D CBCT in comparison to 2D radiographs in the analysis of condylar morphology [8], [9], the segmentation and 3D rendering of condyles using CBCT is still problematic [10]. The diversified and complex morphology of condyles, relatively low condylar bone density, proximity of the discus articularis and the overshadowing glenoid fossa have made the condyles anatomically a challenging structure to depict [11], [12]. In addition, the intrinsic low contrast resolution, partial volume effect and distortion of Hounsfield Units (HU-value) in CBCT scans have hampered an accurate 3D rendering of the condyles from raw CBCT data sets [10], [11], [13] (*Figure 1*).

A wide range of approaches have been developed to render the condylar surface in 3D using CBCT data. In all presented approaches, the 3D surface of the condyles was computed through manual outlining of the condylar contour in 2D cross-sections of a CBCT scan, based on the subjective judgment of an observer to identify the condylar contour. The observer distinguishes the condyle from adjacent tissues through differences in gray-scale value while taking the general shape of condyle into account. This process is tedious and time-consuming. The outlines traced in subsequent slices may become mismatched, resulting in an irregular surface rendering and an erroneous analysis. The semi-automated 3D rendering of condylar surface approach by Paniagua et al. in their SPHARM-PDM analysis provided some promising results and showed an appropriate precision for its clinical precision [14], [15]. However, this approach is dependent on the manual outlining of surface structures in cross-sections of a volumetric dataset with a user-guided InsightSNAP software application [16]. Our previously proposed approach for 3D rendering of condylar surface showed to be highly reliable and reproducible to reconstruct 3D morphology of the condyles [12]. Nonetheless, it had the drawback of necessitating the use of various software applications and manual check and refinement of the thresholded condylar outline on each slice. In order to meet the clinical demand, a more automated approach for the 3D reconstruction of condylar surface with proven reliability and validity and a significantly reduced computing time is desired.

The aim of the current study is to present a fast, semi-automated approach for 3D surface rendering of condyles using CBCT data, and to validate the clinical performance of this method.

## Materials and Methods

The study protocol (number 181/2005) was approved by the Medical Ethical Commission of the Radboud University Nijmegen Medical Centre, Nijmegen, The Netherlands. Patient information was anonymized and de-identified prior to analysis. Due to the design of the study and the usage of anonymized data, written informed consent was waived for this study.

### Patients

CBCT datasets of ten adult Caucasian patients (2 males and 8 females, mean age 38.1 years, range 19 to 58 years), randomly selected from the CBCT database of the Department of Oral and Maxillofacial Surgery, were used for this study.

### Data acquisition

The CBCT datasets were obtained by scanning the patients seated in the natural head position using a standard CBCT scanning protocol (i-CAT, 3D Imaging System, Imaging Sciences International Inc, Hatfield, PA, USA) in “Extended Field” modus (field of view: 16 cm diameter/22 cm height; scan time: 2×20 seconds; voxel size: 0.4 mm) at 120 kV and 3–8 mA pulse mode. Radiation dose for the patient was given as 136 µSv for a single scan.

Two observers segmented a total of twenty condyles using all ten datasets with the semi-automatic segmentation method proposed in the current study. Observer 1 had extensive clinical experience with the segmentation of condyles based on CBCT data, whereas observer 2 had little clinical experience. The segmentation results of both observers were referred to as Segmentation Group 1 (SG1) for observer 1 and Segmentation Group 2 (SG2) for observer 2.

The previous validation study by Xi et al. [12] utilized the same ten datasets as the current study. As the results from the aforementioned study were proven to be highly reliable and reproducible, the segmentations and 3D reconstructions from the previous validation study served as controls in the current study. These controls will hereafter be referred to as the Validation Group (VG).

### Semi-automatic segmentation

The DICOM files were loaded in a Graphical User Interface designed in Matlab (2012b, The Mathworks Inc., Natick, MA, USA) for the visualization and segmentation of an image volume. For means of search region reduction and reduction of computational resources, a smaller volume of interest (VOI) was selected by the observer before initialization of the segmentation. A novel semi-automatic segmentation algorithm, based on 3D region growing, was implemented in the software. A seed point was selected by the observer once every five slides in the VOI. After selection of the seed point, a 2D connected components region growing algorithm provided the observer with an outline of the segmentation. The threshold value of the 2D region growing could be adjusted interactively. The outline was updated in real time accordingly after the threshold adjustment, providing an optimal thresholding based on the outline of the condyle, according to the judgment of the observer.

**Figure 2:**
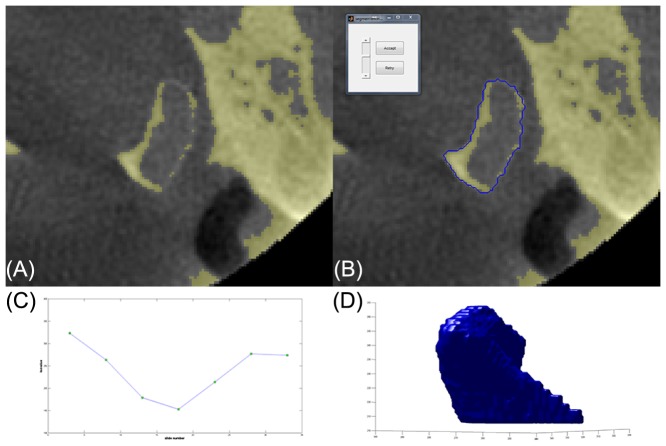
Schematic overview of the segmentation algorithm. The faulty outline of the thresholded condyle is clearly present (A). The user selects a seed-point once every five slides. The selection of this seed point automatically renders an outline of the condyle based on the threshold (B). The user can adjust the threshold interactively to generate the best fit for the condylar outline. All threshold values are plotted in a graph (C). For all slides in between the two user defined slides, the threshold value is determined through interpolation. Subsequently, the region growing is initiated, after which post processing is possible. Finally, the condyle is segmented and rendered in 3D (D).

The selection of local thresholds could be carried out in the axial, sagittal and coronal direction. Following the selection of local thresholds once every five slides, a 3D connected components region growing algorithm that was configured with these local thresholds was initiated automatically from the central seed point. Interpolation was used to establish the local threshold to be used on slides not processed by the user. The algorithm's output consisted of a binary volume which served as an overlay on any of the visualized slides, and a 3D polygon that could be visualized in a separate 3D visualization window. A schematic overview of the segmentation protocol is presented in *Figure 2*.

### Post processing

Post processing of the resulting segmentation was performed using the 2D region growing algorithm mentioned before. A region of interest was selected on a slide in need of correction (applicable in the axial, sagittal and coronal direction). After the selection of a new seed point, a new outline of the condyle was generated, which could again be adjusted interactively. Both addition of a 2D segmentation to the image label already present in the slide from the 3D region growing, as well as replacement of the 3D region growing image label in a particular slide were possible. The polygon surface was updated once the 2D region growing result was accepted by the observer.

In order to counteract possible excess connections between the glenoid fossa and condyle, the segmentation volume could be morphologically opened with a structural element of size three. The segmentation result following post processing was exported as a raw binary dataset which could be processed by Maxilim (Medicim N.V., Mechelen, Belgium) for integration in a 3D virtual model. Since the segmentations were made using the original DICOM data from which the 3D virtual head model was reconstructed, the condyles were reconstructed from the segmentation at the correct position in the 3D virtual head model.

### Statistics

Condylar volumes were calculated for each segmented condyle within the three groups. The statistical analysis was carried out with IBM SPSS Statistics for Windows (IBM Corp., Armonk, NY, USA). A 2-way random-effects analysis of variance (ANOVA) was used to test for errors in the segmentation volume between the three groups (VG, SG1 and SG2). The intra-class correlation coefficient (ICC) was calculated using the volumetric measurements as a measure of conformity between the two observers (SG1 and SG2). A 2-way mixed ICC model on absolute agreement was used. An ICC <0.40 was considered as poor, 0.40–0.59 as fair, 0.60–0.74 as good, and 0.75–1.00 as excellent. A significance level of 0.05 was used for all statistical analysis.

Dice coefficients were calculated for the pairs SG1-VG and SG2-VG in order to assess the accuracy and reproducibility of the currently proposed segmentation protocol. Dice coefficients were also computed between SG1-SG2 to evaluate the inter-observer reliability. The formula for calculation of the Dice coefficient is stated below. Dice coefficients range from zero to one, with zero indicating a complete lack of overlap and one indicating a perfect agreement between the segmentation volumes.
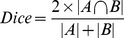



In Maxilim, 3D reconstructions of the segmented condyles were rendered from the binary datasets. Distance maps were computed to assess differences between the contours of 3D rendered condyles. Distance maps between all observers for the segmented condyles (VG -SG1, VG-SG2, SG1-SG2) were generated to determine the error margin at the condylar surface induced by differences in the segmentation procedure. The median, 90^th^ percentile and range were calculated to summarize the surface errors between the three groups.

## Results

The study group consisted of two males and eight females with a mean age of 38.1 years (range 19–58 years). A total of twenty condyles were segmented in each group.

A summary of volumetric measurements of the segmented condyles is given in *Table 1*(see also Table S1). No significant statistical differences in condylar volume were found between the groups VG, SG1 and SG2 (p = 0.96). The mean differences in condylar volume was 18 mm^3^ (1.1 volume-%) between VG and SG1, and 13.4 mm^3^ (0.8 volume-%) between VG and SG2. The mean volumetric difference of condyles between the two observers (SG1-SG2) was 31.4 mm^3^ (1.9 volume-%), resulting in an intra-class correlation of 0.97 within the SG group.

**Table 1:** Descriptive statistics of condylar volume.

Group	Mean (mm^3^)	SD (mm^3^)	Range (mm^3^)
Validation group (VG)	1673.9	343.5	[1011.5–2374.3]
Segmentation group 1 (SG1)	1691.9	337.0	[1043.2–2397.6]
Segmentation group 2 (SG2)	1660.5	316.6	[1095.9–2182.9]

Standard deviation (SD).

An excellent overlap of the segmented condylar surface was found between the three groups. The Dice coefficients for the pairs SG1-VG, SG2-VG and SG1-SG2 were 0.96, 0.96 and 0.98 respectively. The lowest Dice coefficient found in any group was 0.94.

**Figure 3:**
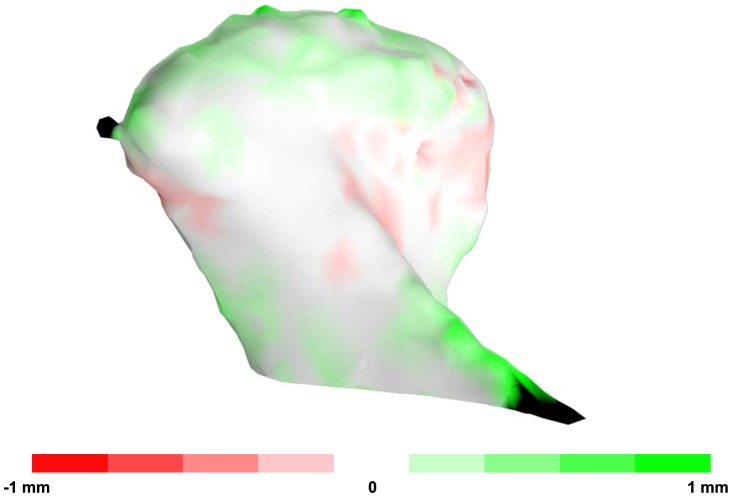
A typical 3D distance map of the manually and semi-automatically segmented condyles. The green area indicates the surface of the manually segmented condyle whereas the red area indicates the surface of semi-automatically segmented condyle. The colour intensity quantifies the distance between both surfaces. Only small differences are present between both surfaces.

The absolute surface errors of 3D rendered condyles were computed from the distance maps (Table S2), and are summarized in *Table 2*. The average median surface distance error induced by the currently proposed condylar segmentation protocol was 0.13 mm, considerably smaller than the voxel size (0.4 mm) and the clinically relevant error margin of 1 mm. A visualization of the surface distance errors between a manual and semi-automatically segmented condyle is given in *Figure 3*.

**Table 2:** Median, 90^th^ percentile and range of surface errors of 3D rendered condyles between the three groups (distance map).

Groups	Average median (mm)	Average 90^th^ percentile (mm)
VG–SG1	0.13 [0.08–0.22]	0.39 [0.22–1.06]
VG–SG2	0.13 [0.06–0.24]	0.37 [0.24–0.98]
SG1–SG2	0.09 [0.05–0.21]	0.32 [0.17–0.72]

## Discussion

The current study showed that the accuracy and reproducibility of the newly proposed semi-automated segmentation protocol of the condyles based on 3D region growing are comparable to the semi-automated segmentation protocols described by previous studies [12], [15]. An excellent inter-observer reproducibility was found for both the 3D surface based measurements as well as for the volumetric measurements.

The major benefit of the current segmentation protocol in comparison to other available segmentation protocols is the significantly reduced image post processing time and improved user friendliness. The average time required to segment the condyle is now approximately ten to fifteen minutes, a four to sixfold decrease compared to the previous study [12]. This significant reduction is achieved due to data interpolation between the slices, eliminating the necessity for the observer to indicate the outline on every single slice, in contrast to the previous study [12]. In the current study, the observer only has to indicate a seed point on 8 to 10 slides in order to generate a primary segmentation of the whole condyle, consisting of 40 to 60 slides. Moreover, in this study, the newly adopted automated post processing steps further reduce the need for manual enhancement after primary segmentation. Moreover, no specific (commercial) software package is required to perform the segmentation procedure, because the graphical user interface built around the segmentation algorithm was exported from Matlab to an executable file (.exe-file), which can be run on any computer or even tablets. This is not only attractive from a cost-effective point of view, but also improves the accessibility of the software, facilitating its implementation in the clinic. In addition, the built-in region growing algorithm and the morphological opening functionality have greatly reduced the amount of manual interactions and errors by the user in segmenting a condyle.

The accuracy and reproducibility of the proposed segmentation process is influenced by random as well as systematic errors. The current study has focused on the quantification of the random error, which is largely attributed to the observer-dependent interpretation error of the condylar outline and variances in the operational procedure. Several previous studies have investigated the observer dependent error. In the study of Paniagua et al. [14], the condyles were segmented manually using the InsightSNAP software. They reported a mean inter-observer distance error of 0.64 mm and 0.68 mm for the left and right condyles. The previous study of Xi et al. [12], in which a less semi-automated segmentation protocol was used, had shown that the mean inter-observer surface error was 0.13 mm. The standard deviation for the volumetric measurements of condyles was 14.16 mm^3^ (0.8 volume-%) using the semi-automated segmentation. In comparison to these segmentation protocols, the random error that is associated with the currently proposed segmentation protocol is even smaller, well below the voxel size of the CBCT scan. This is likely to be the result of the more automated nature of the latter segmentation protocol, which has obviously reduced the magnitude of the observer related errors.

The systemic error can be defined as the sum of errors resulting from 1) the image acquisition, 2) errors yielded by the segmentation algorithm and 3) errors induced by 3D reconstruction of the segmentation.

1. All CBCT scans are affected by image acquisition errors. Besides the obvious influence of the voxel size, several possible image artifacts hamper the spatial resolution in CBCT imaging. Involuntary patient movements during the CBCT scan induce motion related deterioration of spatial resolution. Also, the cone beam geometry hampers the spatial resolution of the condylar region due to a higher noise in peripheral areas of the CBCT scan^18^. Due to the effect of cone beam geometry, the affected voxel will no longer be a representative of an anatomic boundary, but rather a weighted average of the involved structures. This unwanted homogeneity of voxel intensities deteriorates the local spatial resolution and blurs the boundaries between different tissue types, making the segmentation process particularly difficult in the condylar region. By positioning patients in their natural head position, the condyles are in fact positioned adjacent to the horizontal plane of the beam, which reduces the effect of cone beam geometry [17].
2. The built-in morphological opening functionality of the segmentation algorithm may induce systematic errors during condylar segmentation. The intended result of this morphological operation is the separation of two connected objects by the automatic removal of small connections between the condyle and glenoid fossa. However, some of the voxels along the condylar outline may be lost in this process. As only small connections were present at the condylar surface, the opening process could be performed with a small structuring element. This ascertained that only a very small number of voxels in regard to the total condylar volume were involved in the opening process, making the amount of lost voxels negligible.
3. The 3D reconstruction algorithm in Maxilim smoothens the segmentation result, after which the observer chooses a threshold to calculate the 3D reconstruction. The smoothing algorithm could also induce systematic errors to the shape of the segmentation. On the other hand, the smoothed shape will more closely represent the actual shape of the condyle than the “voxelized” segmentation result. By exporting all binary segmentations and reconstructing them with the same threshold, the effect of this systematic error on the results was minimized.

The gold standard used to validate the currently proposed segmentation protocol is manual segmentation. Bearing the possible errors induced by image acquisition in mind, manual segmentation will not be a perfect representation of the “true” shape the observer is trying to segment. Ideally, the morphology of the segmented condyles should directly be measured, for instance with laser surface scanning [18]. While this is not possible in living patients, it can be applied to cadaveric heads in order to obtain a better ground truth measurement. When a better ground truth measurement is acquired, better quantification of the systemic errors of the segmentation methods will be possible.

**Figure 4:**
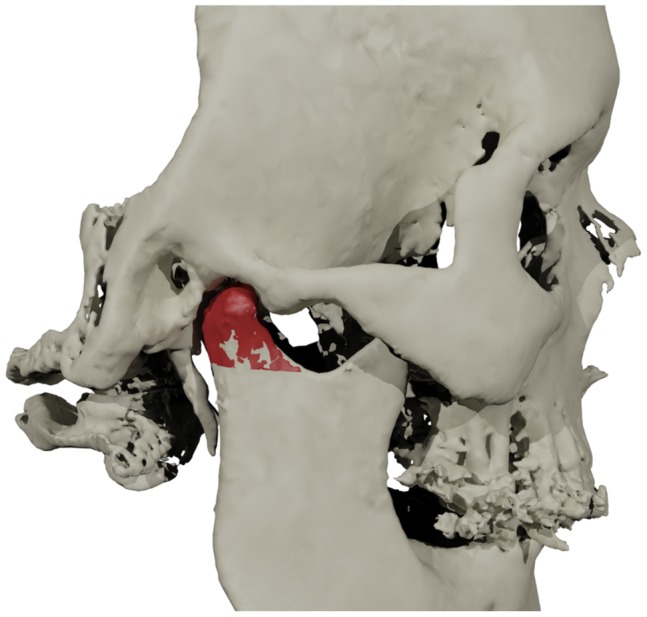
The 3D reconstruction of semi-automatically segmented condyle (red), and its integration into the original 3D virtual head model of **Figure 1**. Condylar measurements can be carried out using this integrated model.

Due to the significantly reduced computing time, the excellent reproducibility and the increased user friendliness of the software, the currently proposed approach for the 3D rendering of condyles can be readily introduced into a clinical setting. The segmentation method allows accurate 3D evaluation of the effects of orthognathic surgery on the condyle (figure 4). Volumetric changes as well as changes in shape over time can now be assessed in 3D, distinguishing PCR from physiological condylar remodeling in an early stage. By the voxel-based superimposition of 3D virtual head models, changes in condylar position (condylar seating) during the course of the combined orthodontic and orthognathic treatment can be evaluated. This complete assessment of the condylar morphology and condylar position in orthognathic surgery will provide sound recommendations to carry out risk assessment for PCR and to indicate further medical interventions to reduce and possibly counteract the unwanted postoperative relapse.

In addition, the currently proposed segmentation protocol can be placed into a broader perspective beyond the field of condylar pathology. The current segmentation protocol can be applied to segment different tissue types, ranging from dentition to soft tissues, by defining a tissue specific seed point accordingly to start the segmentation process. Many anatomic regions of the maxillofacial complex (i.e. maxilla and airway) as well as the results of surgical interventions (i.e. bone grafts) can be segmented and rendered in 3D using the currently developed segmentation protocol. Clinical assessment will thus be possible for these structures of interest, yielding a better understanding of the underlying anatomy and providing a quantification of the possible pathology.

In conclusion, the currently proposed segmentation protocol for the 3D rendering of condyles showed an excellent inter-observer similarity, a drastically reduced computing time and increased user friendliness. The clinical application of this method enables a full 3D follow-up of condylar morphology after orthognathic surgery. The currently proposed segmentation protocol has the potential to be applied to segment different tissue types in various anatomic regions using CBCT datasets.

## Supporting Information

Table S1 **Condylar volumes (mm^3^) obtained by manual segmentation (golden standard), observer 1 (TX) and observer 2 (RS).**
(XLSX)

Table S2 **The mean absolute errors (standard deviation, percentile, range) in condylar volume (mm^3^) between the manual segmented condyles and condyles segmented by both observers using the currently described semi-automated segmentation protocol.**
(XLSX)
